# Contraceptive method use, discontinuation and failure rates among women aged 15–49 years: evidence from selected low income settings in Kumasi, Ghana

**DOI:** 10.1186/s40834-021-00151-y

**Published:** 2021-02-26

**Authors:** Ayaga A. Bawah, Ryoko Sato, Patrick Asuming, Elizabeth G. Henry, Caesar Agula, Charles Agyei-Asabere, David Canning, Iqbal Shah

**Affiliations:** 1 University of Ghana, Legon, Accra, Ghana; 2 TH Chan School of Public Health, Harvard University, Boston, USA

**Keywords:** Contraceptives, Method failure, Discontinuation, Kumasi, Ghana

## Abstract

**Background:**

This paper provides estimates of contraceptive discontinuation and failure rates in a poor urban setting in Ghana. Contraceptive use is for the purposes of preventing unintended or mistimed pregnancies. Unfortunately, evidence abounds in many parts of the world where there is considerable levels of contraceptive failure and high levels of discontinuation resulting in unintended pregnancies.

**Methods:**

We estimated discontinuation rates during a 12-month period since starting use by applying single and multiple decrement life table methods to the contraceptive calendar data collected in a survey of women in reproductive age of 15–49 years.

**Results:**

Modern contraceptive method use was estimated to be 13.7% at the time of the survey. The results show that contraceptive method discontinuation vary markedly by type of contraceptive method but are high for almost all methods, except for implants (23.7%). Discontinuation rate for emergency contraception was estimated at 88.5%, withdrawal 87.6%, and male condom use 80.9%. However, discontinuation rates were moderately high for rhythm (63.6%), pills (65.6%) and injectables (56%). In terms of failure rates, overall contraceptive failure for all methods was estimated at 7.9%. The factors significantly associated with method failure include being within age bracket 40–44 years (OR = 0.3, *p* < 0.05), having secondary/higher education (OR = 0.4, *p* < 0.01), belonging to the richest household wealth scale (OR = 3.3, *p* < 0.01), currently in union with a partner (OR = 2.2, *p* < 0.01), and using contraceptive methods such as rhythm (OR = 5.6, *p* < 0.01) and withdrawal (OR = 3.7, *p* < 0.01). On the flip side, the odds for method discontinuation were significantly higher for women in their 20s and mid 30s, formerly in union (OR = 1.9, *p* < 0.05) and use of withdrawal method (OR = 1.4, *p* < 0.05) and lower for women formerly in union (OR = 0.4, *p* < 0.01) and use of implants (OR = 0.2, *p* < 0.01) and injectables (OR = 0.6, *p* < 0.01).

**Conclusion:**

While contraceptives use is low, both discontinuation and failure rates are high and variable among different methods. Failure and discontinuation rates are lowest for long-acting methods such as implants while higher failure rates are more prevalent among women who rely on withdrawal and the rhythm methods.

## Background

Family planning and contraceptive use remain an effective intervention for addressing women’s reproductive health needs and particularly, preventing unintended and/or, mistimed pregnancies. Indeed, one of the major fertility regulation interventions that received international attention and donor support, particularly in developing countries was the introduction of large-scale family planning programs [1]. Contraceptive use is mainly for the purposes of preventing unintended or mistimed pregnancies [2–4]. Singh, Sedgh and Hussain [4] reported that 40% of an estimated 213 million worldwide pregnancies that occurred in 2012 were unintended. While these rates vary across regions and countries, the highest unintended pregnancy rates are found in Middle and Eastern Africa [4]. High rates of unintended pregnancies are also observed in West Africa where it was reported that 26% of the 17.6 million pregnancies were unintended [4]. In Ghana, about 37% of all pregnancies are also unintended, with 23% being mistimed and 14% being unwanted pregnancies [5, 6].

Unintended pregnancies are a major public health problem which affects both individuals and the society at large. Problems arising out of unintended pregnancies range from health, through emotional and psychosocial problems, as well as economic and in many cases, such pregnancies mark the termination of the educational careers for many girls and women and deprive them of work opportunities, particularly in developing countries.

It is often assumed that women who are using contraceptives are protected from getting pregnant. However, some of the unintended pregnancies happened while the women were reported to be using contraceptives. For instance, in 2008 Kost et al. reported that as much of 12.4% women in the United States of America who were using reversible methods in 2002 had experienced a contraceptive failure after 12 months of initiation of use [7]. Trussell found slightly higher rates (14.9%) using the same dataset [8]. Using data from 43 countries Polis et al. [9] found 12-month failure rate varied across sub-regions and by method. Overall failure rates were lowest for long-acting reversible methods; for example 0.6% for implants, 1.4% for IUD and 1.7% for injectables and highest for traditional methods such as withdrawal or period abstinence (13.9%). More recent results from a paper by Bradley et al. [10] show similar patterns of failure with respect to method, although more generally their rates are higher than those reported by Polis et al. in 2016. For instance, Bradley et al. estimate failure rate for implants as 0.03 (per 100 episodes), that for IUD as 1.2, and approximately 2.0 for injectables. Failure rates are higher for pills (6.3%), condom (8.6% and periodic abstinence 19.0% [10]. What this suggests is that contraceptive failure or user-failure resulting from incorrect or inconsistent method use prevent women from realizing their reproductive intentions.

As noted above, some of unintended pregnancies are a result of contraceptive method failure, which is also one of the reported reasons for method discontinuation. Other often cited reasons for discontinuation are side effects or health concerns and desire to become pregnant or no further need for using a method [3]. Analysis by Ali, Cleland and Shah using data from 60 Demographic and Health Surveys also show that discontinuation rates and reasons for discontinuation, vary by method [3].

The demographic impact of contraceptive use depends not only on its prevalence but also on the duration and effectiveness of use. As desired family size declines and contraceptive prevalence rises, contraceptive effectiveness becomes an increasingly important determinant of fertility [11]. For example, if desired fertility is six births per woman, an increase in contraceptive effectiveness from 85 to 90% would result in a decline in total fertility of about 0.20 births. In contrast, if desired fertility is two births per woman, then the same increase in contraceptive effectiveness would reduce the total fertility rate by more than one birth [12].

In addition to the demographic impact, the analysis of contraceptive discontinuation, and in particular, contraceptive method failure, is important because it can inform efforts to improve service delivery in various ways. For instance, the rate at which women discontinue the use of a method due to reported side effects may signal the need for improved counseling, and that information about the method needs to be communicated much more effectively [13–15]. High levels of discontinuation due to access or availability problems suggest that supply and/or distribution mechanisms need examination. Combined with knowledge of the modes of delivery for different methods, discontinuation rates can help identify the inadequacies of particular types of service delivery and the potential effects of contemplated changes in modes.

This paper reports on estimates of contraceptive method use, discontinuation and contraceptive method failure rates and their determinants in an urban poor setting in the city of Kumasi in southern Ghana. Kumasi was one of two cities in Ghana where Willows International, in partnership with government of Ghana and four other organizations, implemented the *Reducing Maternal Mortality and Morbidity (R3M)* program that aimed to improve access to family planning and safe abortion services [16]. The interventions implemented included a package of reproductive health information programs that focused on provision of reproductive health information and home-based family planning counseling and referral services for women who desire to adopt family planning. Women identified as being at high risk of unintended pregnancy as well as other reproductive health problems were  referred to selected health facilities for services.

## Methods

### Study setting and sampling procedure

This paper is based on data collected in four communities in the Kumasi metropolis as part of a retrospective, cross-sectional survey by Willows Impact Evaluation (WIE) study to evaluate the impact of the Willows program. Data were collected in Willows intervention areas: Asawase-Aboabo and Sepe-Buokrom communities and comparison areas: Angloga and Sepe-Timpom communities. These communities share similar socio-economic, demographic and ethnic mix characteristics and usually vulnerable to the exigencies of urbanization such as floods and cholera and often disproportionately limited in terms of access to health care.

WIE study employed a multi-level cluster sampling procedure for a representative sample. In the first level, 200 clusters (100 each for intervention and comparison areas) were randomly sampled from sub-divisions of Enumeration Area (EA) maps obtained from the Ghana Statistical Service (GSS). A complete listing of all women aged 15–49 years in the sampled clusters was done. In the second level, about 20 households with at least a woman within the age band 15–19 years were randomly sampled from each sampled cluster. In the last level, where there were more women within the age bracket 15–49 years in a household, one was randomly selected.

### Data collection and tools used

A population-based survey was implemented during the period January–July 2018. Data collected included indicators of contraceptive use and method preferences, indices of unmet need, discontinuation and whether or not women got pregnant while using a method. These data were collected using an electronic version of a paper questionnaire with both standard direct survey questions and the use of a retrospective contraceptive calendar covering reproductive events during the 5 years prior to the survey, based on the Demographic and Health Survey (DHS). CommCare version 2.40.1, an electronic data capture system, was used for programming of the paper questionnaire into an electronic version for data collection [17]. CommCare allows for the capture and storage of data in real-time, with features to program inbuilt checks to prevent inconsistent and common errors from getting into the data. It also offers the ability to monitor and control the experiences of data collectors in the field and ensures that interviewers follow strict protocol which helps to reduce errors that commonly get into the data when using manual or paper-based data collection techniques. This paper draws on these data to estimate contraceptive use, discontinuation and failure rates, among all sexually active women who were using contraceptives during the period.

### Measures

Three status indicators of contraceptive use dynamics permit the estimation of contraceptive failure and discontinuation rates. These include getting pregnant while using a method of contraception, timing in switching from one method to another or abandoning the use completely. Contraceptive failure is estimated by calculating the number of observations with reported pregnancy while on a method relative to all who were using the method, while discontinuation rates are estimated as those who stop using a method for whatever reason and duration. Discontinuing the use of a method may or may not be followed by method switching. Method switching occurs when a woman switches from the use of a contraceptive method to another method. Women who switch from one method to another are considered to have discontinued the previous method. The unit of analysis is an episode of contraceptive use, which begins at the point in the reproductive calendar when a woman reports initiating use of a contraceptive method and ends when she reports discontinuing that method [9].

The estimation procedure is based on all women who reported that they were sexually active and had used a method of contraceptive at some point during the period as captured in the contraceptive calendar. The period covered by the contraceptive calendar is 5 years preceding the survey. An individual woman may contribute more than one episode to the calculation during the period. Women who reported that they discontinued the use of any method during the period because they became pregnant while using the method are captured as method failure. This permits the identification of episodes of use and discontinuation.

The analysis is restricted to episodes within the last 5 years preceding the survey using data from the contraceptive calendar. For periods of use between births occurring within the 5 years it is possible to calculate periods of starting dates taking into consideration reported duration of use and nine-month gestational period for more recent births [18].

The study considered the use of methods such as sterilization, intrauterine device (IUD), injectables, implants, pills and male/female condom, emergency contraceptive (EC), lactational amenorrhea as modern methods, consistent with the 2017 Ghana Maternal Health Survey [19]. Withdrawal and calendar/rhythm/periodic abstinence were considered as traditional methods.

Measurements for explanatory variables used in the regression models were: age (1. 15–19 years, 2. 20–24 years, 3. 25–29 years, 4. 30–34 years, 35–39 years and 40+ years), religion (1. Catholic, 2. Methodist/Anglican/Presbyterian, 3. Pentecostal, 4. other Christians, 5. Moslem and 6. other/no religion), educational level (1. none, 2. primary and 3. secondary/higher), household wealth index (1. poorest, 2. poorer, 3. middle, 4. richer and 5. richest), marital status (1. single, 2. currently in union with a partner and 3. formerly in a union with a partner), contraceptive method (1. male condom, 2. rhythm, 3. withdrawal, 4. injection, 5. pill and 6. implant) and knowledge of family planning methods. Women who used EC were dropped from the regression analysis because it is meant for emergency situations and expected to be discontinued after use. These variables were selected based on previous literature [20–22] and other possible reasons that could affect the dependent variables.

### Method of analysis

Discontinuation rates are estimated using single and multiple decrement life table procedures as implemented in the DHS programme [23]. It calculates the probabilities of discontinuation for specific methods in the absence of competing reasons for discontinuation. The procedure calculates percentage of episodes discontinued within 12 months by reason for discontinuation, by method.

To estimate the determinants of both discontinuation and failure rates, we used logistic regression methods, dichotomizing each into occurrence or non-occurrence of the event (*discontinuation* vs *continuation* and *failure* vs *non-failure*). In the first regression model, occurrence of method failure was coded “1” whereas non-failure coded “0”. For the second model, occurrence of method discontinuation was coded “1” and continuous use coded “0”. Covariates of the models with probability values less than or equal to 5% were considered significant. The pseudo-R^2^ and the f-statistics were used to examine goodness of fit and the overall significance of the models, respectively. Multicollinearity was also checked to ensure that the regressors were not correlated. Moreover, STATA 14.2 which was used for the analysis automatically checks for multicollinearity and often deletes variables that are correlated.

## Results

### Background characteristics of women

A sample of 4230 women in urban-poor settings of Kumasi, Ghana consented to participate in the study and had completed interviews. Of the 4230 women, data of 4220 women after cleaning were used for the analysis. Table 1 provides information on the background characteristic of respondents. Majority of the respondents are less than 30 years of age, with the highest concentration in the age group 25–29 (≈21%). With respect to education, 74.2% of the women had at least some education whereas 25.8% had not had any formal education. However, 26.6% of the women had attained secondary or higher education whereas 47.6% had attained only primary education.

**Table 1:** Distribution of women, by background characteristics

Background characteristics	Case [Percent] (*N* = 4220)
Age (years)	
15–19	556 [13.2%]
20–24	730 [17.3%]
25–29	869 [20.6%]
30–34	700 [16.6%]
35–39	610 [14.4%]
40–44	402 [9.5%]
45–49	353 [8.4%]
Education	
None	1089 [25.8%]
Primary	2008 [47.6%]
Secondary or higher	1123 [26.6%]
Household Wealth Index	
Poorest	323 [7.7%]
Poorer	829 [19.6%]
Medium	11,571 [37.2%]
Richer	1230 [29.2%]
Richest	267 [6.3%]
Marital Status	
Currently in union with a partner	1656 [39.2%]
Formerly in union with a partner	2173 [51.5%]
Currently in union with a partner	391 [9.3%]
Religion	
Catholic	237 [5.6%]
Anglican/Methodist/Presbyterian	371 [8.8%]
Pentecostal	1265 [30.0%]
Other Christian	511 [12.1%]
Moslem	1780 [42.2%]
Other/No religion	56 [1.3%]
	Mean [Std. Dev.]
Number of contraceptive methods known (0–13)	8.6 [2.0]

In terms of household wealth of the respondents, about 7.7% of them are categorized as being in the poorest category while 5.3% are in the richest category. The greater proportion of them are either in the medium or richer categories. Majority (37.2%) of the respondents are in the middle category.

With regards to marital status, 39.2% of the respondents were reported to be single, whereas 51.5% were said to be in a union with a partner at the time of the survey. About 9.3% of the respondents reported that they were formerly in a union with a partner. With respect to religious affiliation, most of the respondents professed as being Christians (56.5%) with majority of the Christians being Pentecostal (30.0%). However, a large proportion of them  also  reported as being Moslem 42.2% and a small minority of 21.3% indicating no religion.

In terms of knowledge of contraceptive methods, respondents had knowledge of an average of 8.6 methods out of a total of 13.

### Method mix for women

**Fig. 1:**
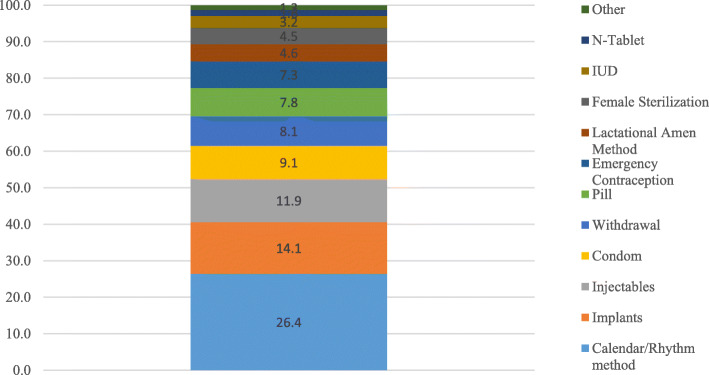
Method Mix for Women 16–49 in Kumasi, Ghana 2018

Figure 1 shows method mix for women. The results show a broad mix of various contraceptive methods that women in this area use for fertility regulation. Overall, modern contraceptive prevalence rate in this population was estimated to be 13.7% at the time of the survey. However, as Fig. 1 suggests, women in these communities use both modern and non-modern methods of contraceptives for purposes of fertility regulation. The majority of contraceptive users use calendar/rhythm method which is a traditional method (26.4%), followed by implants (14.1%), then injectables (11.9%). Other modern methods that women use include male condom (9.1%), emergency contraception (7.3%), female sterilization (4.5%), IUD (3.2) and lactational amenorrhea method (LAM) (4.6%). Another traditional method commonly used was the withdrawal method which is reported to be 8.1%. A few of the women (1.6%) also reported using Primolut N-tablet (commonly referred to as N-tablet in Ghana). The N-tablet contains norethisterone and is often used as a pre- or post-coital tablet by women to prevent pregnancy.

### Method discontinuation and method failure by type of FP

The first part of Table 2 reports results for 12-months discontinuation rates for women by method and reasons for discontinuing use of particular methods. The first part of the table represents episodes of use. Rates vary markedly by method but are overall high for almost all methods, except for implants (23.7%). As expected, the rates were very high for EC (88.5%), withdrawal (87.6%) and male condom use (80.9%). The rates were however moderately high for rhythm (63.6%), pills (65.6%) and injectables (56%).

**Table 2:** Method discontinuation and failure rates by type of FP

Contraceptive method	Reasons for discontinuation (%)									Prevalence of Method Failure by type of FP		
	Method failure	Desire to become pregnant	Other fertility related reasons (infrequent sex, husband oppose, fatalistic)	Side effects or health concerns	Wanted more effective method	Other method related	Other/DK	All reasons	Number	Number of episodes	Number of failures (failure rate (%))	% of total failures
Rhythm	6.6	5.5	36.6	0.0	10.3	1.0	3.5	63.6	964	740	106 (14.3)	45.5
Emergency Contraception	4.0	3.0	55.6	12.7	2.8	4.2	6.2	88.5	620	560	27 (4.8)	11.6
Withdrawal	7.7	7.3	45.7	0.3	19.8	1.7	5.1	87.6	619	550	50 (9.1)	21.5
Male condom	2.1	5.6	50.1	1.8	7.0	7.0	7.4	80.9	532	454	11 (2.4)	4.7
Injectables	2.5	9.4	8.7	25.7	0.9	3.1	5.8	56.0	360	258	12 (4.7)	5.2
Pills	5.7	8.1	19.3	20.3	4.0	4.3	4.1	65.6	343	273	25 (9.2)	10.7
Implants	0.5	2.5	2.1	16.6	0.0	0.0	2.0	23.7	228	104	2 (1.9_	0.9
All methods/Total	4.8	5.8	36.9	7.8	8.1	3.0	5.0	71.4	3666	2939	233 (7.9)	100.00

In terms of reasons for discontinuation of specific methods, the most frequently cited reason for discontinuing the use of implants is side effects/health concerns. The methods mostly discontinued by women due to method failure are the rhythm method (6.6%) and withdrawal (7.7%). Interestingly, the most cited reason for discontinuation for most of the methods is either infrequent sex, opposition from husband or some fatalistic reason.

The second part of Table 2 presents results of contraceptive failure rates. The overall contraceptive failure rate is 7.9%, with rhythm and withdrawal methods recording the highest failure rates. It is important to note the difference between the failure rate for all methods reported in the two divides of Table 2. The first part of Table 2 include all episodes whereas the second part include episodes that discontinued which accounts for differences in the denominators.

### Associated factors of method failure and discontinuation

Table 3 presents logistic regression results showing the determinants of method failure and method discontinuation. The regression for the determinants of method failure is based on those who discontinued use because of method failure relative to all users while that for discontinuation is based on all discontinued events i.e. all who discontinued for whatever reason.

**Table 3:** Logistic regression of associated factors to Method Failure / Discontinuation

Indicator Variable	Method Failure				Discontinuation			
Age Category	Odds ratio	***P***-value	95%-CI		Odds ratio	P-value	95%-CI	
RC (15–19)								
20–24	0.54	0.19	0.22	1.35	1.89	0.00	1.23	2.92
25–29	0.96	0.93	0.39	2.39	2.44	0.00	1.55	3.84
30–34	0.71	0.48	0.27	1.88	1.67	0.03	1.05	2.67
35–39	0.69	0.48	0.24	1.94	1.59	0.06	0.97	2.61
40–44	0.29	0.05	0.08	1.00	1.65	0.08	0.93	2.92
45–49	0.11	0.05	0.01	1.02	1.72	0.18	0.76	3.87
Religion								
RC (Catholic)								
Anglican/Methodist/Presbyterian	1.70	0.23	0.71	4.09	1.05	0.84	0.64	1.72
Pentecostal	1.42	0.36	0.66	3.06	1.33	0.16	0.89	2.00
Other Christian	1.55	0.32	0.65	3.69	0.75	0.21	0.49	1.18
Muslim	1.08	0.83	0.50	2.34	1.48	0.05	0.99	2.22
Other/No religion	0.86	0.86	0.15	5.01	1.33	0.50	0.58	3.03
Education								
RC (No Education)								
Primary	0.71	0.16	0.44	1.15	1.08	0.55	0.84	1.38
Secondary or higher	0.44	0.00	0.24	0.80	1.18	0.28	0.88	1.61
Household wealth index								
RC (Poorest)								
Poorer	1.61	0.24	0.72	3.60	0.45	0.00	0.31	0.65
Medium	2.00	0.05	1.00	4.03	0.66	0.02	0.46	0.94
Richer	1.72	0.15	0.82	3.60	0.69	0.04	0.48	0.99
Richest	3.32	0.00	1.36	8.10	0.51	0.01	0.30	0.88
Marital Status								
RC (Single)								
Currently in union with a partner	2.18	0.00	1.40	3.40	0.45	0.00	0.34	0.58
Formerly in union with a partner	0.94	0.88	0.41	2.17	1.95	0.02	1.10	3.46
Contraceptive method								
RC (Condom)								
Rhythm	5.63	0.00	2.88	11.00	0.65	0.00	0.47	0.88
Emergency Contraception#	.				.			
Withdrawal	3.71	0.00	1.84	7.47	1.43	0.04	1.01	2.02
Injection	1.12	0.80	0.43	2.93	0.58	0.00	0.41	0.83
Pill	2.85	0.00	1.30	6.28	0.90	0.59	0.62	1.31
Implant	0.39	0.24	0.08	1.92	0.19	0.00	0.13	0.28
Knowledge of number of FP methods	1.10	0.17	0.96	1.27	1.03	0.46	0.95	1.10
Constant	0.00	0.00	0.00	0.03	3.85	0.00	1.71	8.65
	*Number of obs. = 2378; Wald Chi2(25) = 115.3; Prob > Chi2 = 0.00; Pseudo R2 = 0.12; Log pseudolikelihood = − 625.7*				*Number of obs. = 3043; Wald Chi2(25) = 288.75; Prob > Chi2 = 0.00; Pseudo R2 = 0.10; Log pseudolikelihood = − 1428.6*			

#_Emergency contraceptive was dropped from the regression analysis because it is meant for emergency situations and expected to be discontinued after use

For failure, the significant determinants are age of woman, education, household wealth scale, marital status, and contraceptive method type. Similarly, apart from education, the rest of the other variables mentioned above are also significantly associated with discontinuation. For instance, women who have secondary or higher levels of education are 0.4 times as unlikely to experience method failure relative to those without education. Also, women within the age band 40–44 years have lower odds (OR = 0.3, *p* = 0.05) for method failure compared with women aged 15–19 years. Surprisingly, women who are better off on the household wealth scale are more likely to experience method failure relative to those in the poorest class, controlling for the type of method used. For example, those in the richest wealth scale are 3.3 times as likely to experience method failure juxtaposed with those in the poorest wealth quintile, and this statistically significant at 1%. Women who are currently in union with a partner (OR = 2.2, *p* < 0.01) are more likely to experience method failure relative to those not married. Unsurprisingly, the likelihood of method failure is higher among methods considered less effective (rhythm and withdrawal) compared with condom.

With regards to method discontinuation, age is significantly associated with method discontinuation. Women within the age band 20–29 years have higher odds of discontinuation relative to younger women (16–19). For instance, comparing with aged 15–19 years, women within the age group 25–29 years are 2.4 times as likely to discontinue the use of a method. Further, results show that women within the richer (OR = 0.7, *p* < 0.05) and richest (OR = 0.5, *p* < 0.05) wealth scales have lower odds relative to their counterparts in the poorest wealth scale. Unlike method failure, women who reported currently being in union with a partner (OR = 0.4, *p* < 0.01) are less likely to discontinue use of a method relative to women who are single. On a flip side, those formerly in union are about 2 times as likely to discontinue use of a method compared with single women. The likelihood of method discontinuation is also lower for the use of rhythm (OR = 0.6, *p* < 0.01), injectable (OR = 0.6, *p* < 0.01) and implant ((OR = 0.2, *p* < 0.01) and higher for withdrawal (OR = 1.4, *p* < 0.05).

**Fig. 2:**
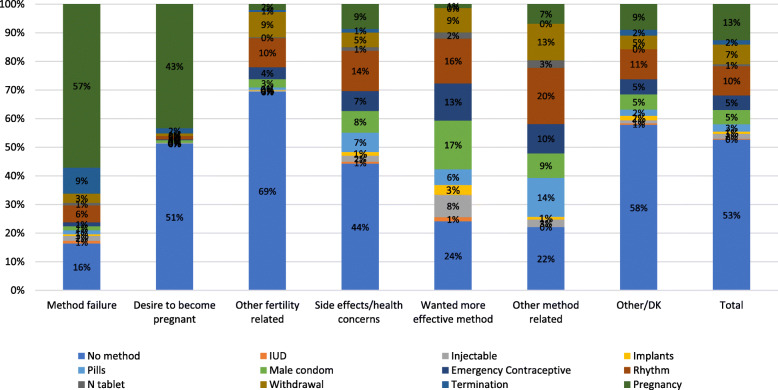
Events after Discontinuation (in 3 months)

Figure 2 shows the reproductive statuses of women 3 months following discontinuation of a method for any reason. Majority of women who reportedly discontinued a method as a result of failure remained pregnant (57%) 3 months following the discontinuation. About 9.1% of the women who reported method failure as the reason for discontinuation terminated the pregnancy. The remaining 16% of the women who reported no method are likely to still be pregnant since they reported a method failure.

For those who desired to become pregnant, the 51% of them did not use a method following discontinuation. Indeed, about 43% of these women became pregnant 3 months following their discontinuation of the method.

## Discussion

### Contraceptive use, discontinuation and failure rates in low-income urban settings

Modern use of contraceptives in low-income urban settings in Southern Ghana is extremely low, about half the national average (25%) [19]. While contraceptives use is low, discontinuation rates are quite high and variable among different methods. Discontinuation rates are lowest for long-acting methods such as implants while failure rates are more prevalent among women who rely on withdrawal and the rhythm methods.

It is not surprising that the rhythm and withdrawal methods have relatively high failure rates because they are entirely under user control with high risk of inconsistent and incorrect use. On the other hand, as expected, failure rates are lowest among implant users. Surprisingly, we find relatively high rates of failure among women who use injectables and oral pills which are considered as relatively more effective compared to condoms or emergency contraceptives, though all depend on user’s correct use to be effective.

With such low use of contraceptives and high discontinuation and failure rates, it is likely that women in these communities will be predisposed to various reproductive health challenges particularly those related to unwanted and mistimed pregnancies, as observed in other parts of Africa and elsewhere in the developing world. Indeed, research published in 2019 [5] using data from the 2014 Ghana Demographic and Health Survey showed that 40% of pregnancies were unintended. Our own data used for this paper show that 33.9% of the women reported that their last pregnancy was not intended.

### Determinants of method failure and discontinuation

Contraceptive method failure is one of the reasons for contraceptive discontinuation. Results suggest that women who use methods such as rhythm, withdrawal and pills are more likely to experience method failure. This is interesting because these methods are often unreliable because of the difficulty in their administration and operationalization. Indeed, Polis et al. have shown that the rhythm and withdrawal methods have the lowest success rates. Results also suggests that the chances of women in their 40s and older to have had experienced method failure is low. Most women beyond 40s are either about entering to, or already in their menopausal ages and would have minimal chances of conception. It is therefore unclear whether the lower odd for method failure is as a result of their fertility chances or other factors such as experience in the proper use of methods overtime. Women with tertiary or higher formal education are associated with lower odds of method failure. This outcome is expected because these women often tend to have more knowledge of contraceptive methods and are more inclined to use effective or modern methods which have less risk of failure. In fact, a plethora of studies in Ghana (e.g., [24–26]) have shown that higher education is associated with the use of effective contraceptive methods. Further, women who reported currently in union with a partner have higher odds of method failure relative to those who are single. It is likely to be the case that single women will take extra care to use more effective methods because they may not want to get pregnant.

Regarding factors associated with method discontinuation and not related to failure, we found that women in their 20s were more likely to discontinue use in low-income settings of Ghana. This outcome contrast with findings for the whole country [22]. Modey and colleagues, using data from the Ghana Demographic Health Survey, 2008 showed that age was not a predictor of method discontinuation. However, our finding is expected because younger aged women are associated with highest levels of fertility. In other words, women in these groups are more likely to have begun active childbearing and so are more likely to discontinue use if they were previously using any contraceptives.

This study is limited in a few ways. The population surveyed is not representative of the entire population of the Kumasi metropolis. The communities selected for this study are low income communities, predominantly of migrant populations and extremely poor with low educational attainment. Secondly, calendar method by which these data are collected is likely to introduce recall biases because of the retrospective interviewing that is associated with the calendar method.

Also, using episodes of use as the main unit of analysis is not straightforward because one could potentially have several episodes of use within the period in consideration.

## Conclusion

Our results highlight the profound program challenges in maintaining contraceptive uptake because of high failure and discontinuation rates in this urban poor setting. The combination of low contraceptive use with high discontinuation and low switching to more effective methods require urgent attention of policy makers and program managers to address the gaps in the provision of counselling and provision of services. The implications of the high discontinuation and or, failure rates suggest that women must be counselled properly on the various methods of contraceptive, their effectiveness and side effects to enable women make appropriate choices to reduce high rates of discontinuation and failure.

## Data Availability

Dataset will be made available by the corresponding author upon reasonable request.

## Abbreviations

DHS: Demographic and Health Survey
EA: Enumeration Area
EC: Emergency Contraceptive
GHS: Ghana Health Service
GSS: Ghana Statistical Service
IUD: Intrauterine Device
LAM: Lactational Amenorrhea Method
R3M: Reducing Maternal Mortality and Morbidity
WIE: Willows Impact Evaluation
